# Navigating Medical Curriculum Renewal: Faculty Experiences of Implementing a Renewed Assessment Plan Through the Lens of Change Theory

**DOI:** 10.1007/s40670-025-02480-y

**Published:** 2025-08-20

**Authors:** Rhoda Meyer, Karin Baatjes, Liezl Smit, Elize Archer

**Affiliations:** 1 Faculty of Medicine and Health Sciences, Department of Health Professions Education, Stellenbosch University, Parow, South Africa; 2 Division of Surgery, Faculty of Medicine and Health Sciences, Stellenbosch University, Parow, South Africa; 3 Faculty of Medicine and Health Sciences, Department of Paediatrics and Child Health, Stellenbosch University, Parow, South Africa

**Keywords:** Curriculum renewal, Medical education, Faculty development, Assessment renewal, Change leadership

## Abstract

Curriculum renewal in medical education has received significant attention in recent years. While contemporary literature has highlighted various recommendations to guide curriculum renewal, an important perspective remains underexplored—the experiences of faculty who are responsible for implementing these changes. In this article, we provide insights into faculty’s experiences of the change process as they implement a renewed assessment plan for a medical programme, offering some recommendations for faculty development. A qualitative interpretivist approach was used. Focus group discussions were conducted with faculty who are teaching and assessing students on the first three modules in the renewed medical curriculum. A three-tiered approach suggested by Miles and Huberman guided the analysis of the data. Coding was inductive and thematic. Four themes were identified, namely the nature of change, a commitment to changing the purpose of assessment, the need for collaboration in taking the plan forward and the need for a culture that supports learning. The findings suggest that while there are various challenges associated with the implementation of the renewed assessment plan, faculty were generally invested in the process. Furthermore, there is a distinct call to advance faculty development to support skills required to undertake assessment renewal.

## Introduction and Background

The imperative to renew and reform curricula has been a topic of interest over the last decade in medical education [1, 2]. These calls have been in response to various forces influencing medical education, including changing healthcare systems, a shift in the expectations of healthcare professionals’ competencies and the question as to whether programmes prepare students adequately for clinical practice [3]. In response to this, many medical education institutions have been undergoing curriculum renewal processes to prepare their graduates appropriately [4]. Central to these transformations is the renewal of assessment practices, which play a crucial role in shaping students’ learning experiences, ensuring competence and ultimately influencing patient care [5].

Traditional assessment methods in medical education have been criticised for their overemphasis on knowledge recall and their limited ability to assess critical thinking, clinical reasoning and professional competencies [6]. There has been a paradigm shift towards competency-based medical education (CBME), which necessitates more authentic, integrated and longitudinal assessment approaches [7]. In addition, there are calls for a more flexible assessment approach where assessment decisions are based on multiple formative assessments after a meaningful accumulation of data is gathered. Moreover, feedback is seen as an integral part of assessment, provided to the student after each assessment [8].

In keeping with the trends on curriculum renewal, Stellenbosch University underwent a curriculum renewal process of the MBChB (6-year undergraduate medical) programme. Stellenbosch University is situated in a resource-constrained setting, which poses several unique challenges. For example, most faculty working and implementing the renewed curriculum are not specifically appointed to teach on this programme, but have other primary responsibilities, e.g. as clinicians, as lecturers in other departments or as biomedical scientists. In addition, these same faculty are concurrently teaching and assessing students in the outgoing curriculum, adding additional complexity to the situation.

An extensive planning process was undertaken to develop the renewed curriculum, which included a new assessment plan. In the previous curriculum, modules were organised around traditional disciplines, with students learning anatomy, physiology and other subjects in separate modules. The renewed curriculum adopts an integrated model where module content draws from multiple disciplines simultaneously. In terms of assessment, the previous plan consisted of various formative assessments throughout the year, with one summative or high-stakes assessment at the end of the module/year. In addition, there was little focus on feedback after each assessment, an aspect that has taken priority with the new assessment plan. The new assessment plan, aligned with the assessment policy of the institution, was based on a more flexible approach, shifting the focus from a single high-stakes assessment at the end of the module to various low-stakes assessments, utilising assessment for learning as a core principle [6]. The assessment policy seeks to promote ‘flexibility in applying the guidelines and principles for excellent practice regarding assessment’ [9].

While flexible assessment has been shown to have a positive impact on students’ learning [10], its success is dependent on those implementing the assessment. Furthermore, assessment renewal represents a complex change process that demands a shift in faculty’s practices and beliefs [11, 12]. Although significant attention has been paid to the design of assessment programmes [5, 13], less is known about how faculty who are responsible for translating these plans to practice navigate the transition from traditional to renewed assessment practices, especially during assessment reform.

McLeod and Steinert share these sentiments, suggesting that the successful implementation of any curriculum is dependent on how well it is accepted and valued by the stakeholders involved [14]. Harris adds to this, arguing that recommendations for curriculum renewal in medical education are typically not implemented due to a lack of understanding of the curriculum, resulting in a lack of commitment from critical stakeholders of the curriculum, including faculty [15]. In the context of this study, the new assessment plan required an extensive amount of work from faculty, and although they had been teaching on the MBChB programme for several years, this assessment plan was markedly different from the previous plan. Since the assessment plan is integrated into the renewed curriculum as a phased approach, there are likely to be opportunities for refinement to this programme. Thus, in this study, we take a step back to look at assessment renewal as a proxy, focusing on change and the way faculty take up change during curriculum renewal. Through the exploration of faculty’s experiences, we aimed to uncover the challenges, adaptive strategies and faculty development needs of faculty as they navigated the transition from a traditional to a more flexible assessment plan. Understanding these needs is essential for developing targeted faculty development initiatives that can better support the successful implementation of assessment or curricula reforms in medical education and across the higher education landscape [11].

Given that faculty on the ground are the ones implementing curriculum renewal and are thus the leaders of curricular change, we considered Fullan’s framework for change as a lens for this study [16]. Fullan has written extensively about the role of change leaders in driving and sustaining curriculum renewal, emphasising that both the skill and a commitment to change are needed. In his framework, Fullan highlights five components that characterise leaders of change in the knowledge society [16, 17]. These include having a moral purpose, understanding the change process, building relationships, knowledge creation and sharing, and coherence making. While some individuals may develop these characteristics through experience and role-modelling, others will require support in the form of faculty development to acquire the attributes necessary to implement and drive change.

Fullan’s framework has been used in studies related to curriculum renewal across secondary and higher education [18]. While it has not been used as extensively in medical education, many of these ideas can be applied to this field. In addition, although this framework has been used for leaders at higher levels, we share Fullan’s sentiments that curriculum renewal requires leadership at all levels—from top administrators to teachers in the classroom [16, 19, 20].

Since faculty who are involved in curriculum implementation can be seen as those leading change, they would have certain characteristics that inform the way this change takes place. We believed that exploring their experiences would help us understand how they navigate change and deal with the challenges of implementing the renewed assessment plan. Thus, it is not only about the scholarship of teaching, learning and assessment, but also about the development of faculty as those leading and sustaining change. This will build on the curriculum renewal literature related to change, leadership and faculty development. This research thus questions: What are the experiences of faculty with the implementation of the new assessment plan of the renewed MBChB curriculum at Stellenbosch University?

## Materials and Methods

A qualitative interpretive study was undertaken that allowed exploration of the subjective meanings and interpretations participants assigned to their experiences. Since we wanted to understand the experiences of an aspect of the curriculum renewal process, purposive sampling was used, where all 29 faculty who taught and assessed students in the first three modules (18 months of the renewed curriculum) of the MBChB programme were contacted by email with the information and informed consent form (Table 1). They were asked to contact the research team should they wish to participate. As mentioned in the introductory section, these faculty have other primary roles, such as teaching on other programmes, resulting in them having to juggle various responsibilities, and in some cases, concurrently facilitating two different curricula for the same programme.

**Table 1:** Modules in the renewed MBChB curriculum

Year	Modules
*Year 1: semester 1*	*Being and Becoming in Healthcare (Part 1)*
*Year 1: semester 2*	*Form and Function*
*Year 2: semester 1*	*Health and Wellness*
Year 2: semester 2	Dysfunction
Year 3: semester 1	Medical Detective
Year 3: semester 2	Interventions
Years 4 and 5	Clinical Curiosity
Years 4 and 5	Being and Becoming in Healthcare (Part 2)
Years 4 and 5	Clinical Rotations Compulsory & Elective
Year 6	Distributed Apprenticeship

Modules in italics are those included in this study

Data was generated by means of focus group discussions (FGDs), responding to the suggestion by Novak et al., who argued that research on curriculum renewal should focus on qualitative data generation with appropriate role-players to highlight the implementation challenges associated with the outcome of curriculum renewal [3]. The choice of FGDs was based on several considerations. Firstly, it allows for the exploration of collective understanding and experiences related to assessment renewal. In addition, the interactive nature of FGDs can stimulate discussion and generate insights that might not emerge in individual interviews [21]. A semi-structured interview schedule, informed by the literature, guided the discussions. This interview schedule included questions such as ‘how would you describe ‘change’ in assessment practices as it refers to the renewed curriculum?’ and ‘what do you think are important considerations with respect to curriculum change, specifically the assessment plan?’.

We initially planned two focus groups to ensure representation across different modules in the programme. Out of the total of 29 faculty teaching on these 3 modules, 10 agreed to participate (see Table 2 for the years in which they taught and for their roles). Two FGDs were conducted in person with these faculty. FGD 1 consisted of 6 participants, and FGD 2 consisted of 4 participants. The FGDs, which ranged from 45 to 60 min, were audio-recorded and transcribed verbatim by a transcriptionist. After transcript preparation, which involved verifying transcripts for accuracy against recordings and removal of identifiers [22], qualitative data analyses were undertaken.

**Table 2:** Characteristics of participants

Participant	Year	Role
Participant 1	Year 1	Lecturer
Participant 2	Year 1	Lecturer
Participant 3	Year 1	Clinician
Participant 4	Years 1 and 2	Lecturer
Participant 5	Year 1	Clinician
Participant 6	Year 1	Clinician
Participant 7	Year 2	Scientist
Participant 8	Year 2	Clinician
Participant 9	Year 2	Scientist
Participant 10	Year 2	Scientist

Thematic analysis was undertaken, guided by the research question and the literature review. A three-tiered approach suggested by Miles and Huberman guided the analysis of data [23]. AtlasTi, a qualitative data analysis software, was initially used to code and analyse the data. The first level involved summarising and packaging the data, which included inductive analysis through open coding. This was first conducted independently by each researcher. A consultation was then held with the research team to discuss the various interpretations of the data and to finalise the codes. The next level involved repackaging and aggregating the data. This was also conducted through discussion and deliberation between the research team to identify the categories and themes. The third level involved identifying explanations for the results across the data sets. Data analyses were thus conducted by all members of the research team.

### Trustworthiness

Lincoln and Guba suggest four criteria for trustworthiness, including credibility, transferability, dependability and confirmability [24]. To maintain credibility, the research team spent a prolonged time in the field to understand the context and build trust with the participants. Transferability was achieved by providing a detailed description of the context and the research process. To ensure dependability, the team maintained detailed records of the research process, including raw data and analysis notes. We also remained reflexive through the process. Confirmability was achieved through critically examining our own preconceptions and how these might have influenced the research. This was discussed during regular meetings with the team.

### Ethical Considerations

The study was approved by the Stellenbosch University ethics committee (ID, REC: SBE-2022–26185). Several ethical considerations applied to this study, including the right to informed consent, privacy and confidentiality, self-determination, and justice. After having received all necessary information relating to the study, faculty who eventually agreed to participate were asked to read and sign the consent form. They were also informed that they have the right to withdraw at any time should they wish to do so. No names of participants were reflected in the FGDs, and participants’ responses were identified as respondents P1, P2, P3, etc.

## Results

Data analysis resulted in the development of four interrelated themes, encapsulating faculty’s experiences with the renewed assessment plan (Table 3). These themes include the nature of change, a commitment to changing the purpose of assessment, the need for collaboration in taking the plan forward and the need for an assessment culture that supports change.

**Table 3:** Themes and sub-themes

Themes	The nature of change	A commitment to changing the purpose of assessment	The need for collaboration in taking the plan forward	The need for an assessment culture that supports change
Sub-themes	Change is a complex process	Embracing the change process for the purposes of improving learning	Changes to assessment plans require collaboration	The assessment change process is influenced by the organisational culture
	Change is a process that requires careful consideration	Acknowledging the implementation dip	Collaboration is needed to find collective meaning	A supportive environment enhances response to change
	Challenges drive creativity	Actions driven by a higher purpose	Motivation of self and others is required for successful change	Change in the assessment plan has a positive influence on teaching, learning and assessment culture
	Change must be grounded in theory	Learning is prioritised		A supportive culture means more support in the form of peer feedback and faculty development
	Critical reflections on current practices are necessary to identify gaps	Change must include the exploration of best practices in assessment		The assessment culture can be enhanced by faculty taking ownership for the cultural shift

### The Nature of Change

The nature of change captured faculty’s understanding of the assessment renewal process, highlighting their perspectives on the characteristics and trajectory of the change they were experiencing. Change was described not as a singular, unanimous concept, but as a multifaceted process, reflected in the different ways it was described. Some described the renewal of the assessment plan as a change in philosophy:

I think the philosophy of this renewed curriculum was supposed to be trying to get the students away from marks-driven, focussed on very competitive, always being the best, getting the highest marks, to let them understand that acquiring the knowledge to become a good doctor is actually more than just mark-driven (FGD2: P2).

Others saw it as more practical, suggesting that change needs to be contextual:

Because they were being taught and examined on things that they were highly unlikely to see, and the focus was not necessarily on everyday presentations (FGD2: P4).

In addition, participants spoke about the larger curriculum change as a process, which is complex and imbued with multiple challenges, such as a lack of time and high workloads. These challenges appeared to have shaped their experiences and perceptions of the assessment component of the curriculum, potentially influencing their receptiveness, engagement and adaptability to the change:

When we are approached for upcoming modules, it’s already such a heavy burden to be developing the new work, because if you’re doing this correctly, you can’t just take your old work and offer it as a podcast. You are re-developing your own curriculum for medicine, which is a huge undertaking (FGD2: P1).

It seemed that the actual implementation had brought specific aspects of feasibility to the fore. As a result, many argued for careful planning before the process of assessment renewal, with consideration given to the context and support required for such an endeavour:

But for that, you must then have a plan in place, and there must be buy-in, and there must be support for that (FGD2: P1).

Conversely, one participant indicated that the challenges she experienced forced her to find creative ways to deal with the new assessment plan, such as group OSCEs, making the experience more rewarding:

So, I think the delay in assessing clinical skills a year later is something that we needed to first accept, because that’s not our traditional way of assessing skills…with the group OSCE, we were not sure of how it’s going to pan out, and what we were going to measure (FGD 1: P3).

There were some suggestions that change should be guided by a theoretical foundation and based on evidence:

Being able to have that sort of insight now into the changes that are happening and seeing this is all evidence-based, and it’s based on the best current literature (FGD1: P2).

There seemed to be a conceptual shift experienced by faculty regarding the change in the assessment plan. Initially, they held the belief that the existing assessment plan was grounded in evidence. However, through their active involvement in the curriculum renewal process, they were exposed to new knowledge and theories on assessment. This was manifested in the request for critical reflection on both past and current practices related to validity in assessment, to identify potential gaps in assessment practices and to find ways to fill these gaps:

And then reflecting on how we were assessed 20 years ago, and thinking it’s now so invalid (FGD 1: P2).

### A Commitment to Changing the Purpose of Assessment

From the data analysis, there was a sense that faculty was committed to the new assessment plan as they were willing to work through the initial challenges. This commitment was specifically focussed on changing the purpose of assessment. However, they were not always sure that they had achieved what they envisaged:

I’m not sure that has worked very well, for a number of different reasons (FGD1: P4).

Some acknowledged that there would be teething problems; in other words, there would be an implementation dip, but things would get better:

It’s too early. We couldn’t see anything. We are going to start a study now to compare it, but if there’s time (FGD1: P1).

Furthermore, there was some consensus that this change was necessary, with the idea that the previous assessment plan was not supportive of students’ learning:

I think the assessment was done in isolation by the various divisions or disciplines, and there was over-assessment. There was too much assessment, too much high-stakes assessment, and it wasn’t clearly mapped (FGD 2: P3).

Some participants’ responses also suggested that their actions in terms of assessing students were driven by a higher purpose, which was for the improvement in students’ learning:

I think there were very valid reasons for having to change the curriculum, and assessment was a prime example of it (FGD2: P3).

Thus, throughout this whole process of change, there was an understanding that learning must be prioritised:

I think in a way it should be easier for students, because the whole idea was that we should make it easier for them to learn and to engage with assessments, and I think we’ve done that (FGD2: P2).

The need to explore best practices in terms of this change was suggested, further highlighting participants’ commitment to making the renewed assessment plan a success:

Looking at the research that’s been done, and changing health professions education in general, there are many new approaches. I think it was identified that we probably need to re-look the way that we’re presenting the current curriculum (FGD2: P1).

### The Need for Collaboration in Taking the Plan Forward

Participants indicated that while there is a certain degree of collaboration, more is needed for the renewal process to be successful. They spoke of the existing networks across module teams, and between module chairs and faculty:

In the renewed curriculum, all the disciplines, or most of the disciplines, are involved in a module, and that means that the assessment of that module also needs to come from many different stakeholders (FGD2: P2).

What is lacking is a more formalised plan to find collective meaning across teams and to encourage commitment to more innovative ways of implementing the plan:

If you look at the literature behind curriculum development, it’s important that there are a number of parties involved, one is the clinician, then it’s the educator, the student and then faculty as well (FGD1: P5).

Another finding was the way in which participants responded to challenges related to the assessment plan, in terms of a collaborative effort. Some spoke of the need to motivate themselves to keep going, while others felt that there was a need to draw others in—in other words, to appreciate what others bring to the team, and to find ways to work together to accomplish the goals of the renewed assessment plan:

You must just pull them into your module, first of all on a micro level, and that’s what we’re trying to do for the module that I’m working on now, is in the module team, just make sure that you have a large representation of clinicians, from the word go (FGD1: P6).

### The Need for an Assessment Culture that Supports Change

The participants’ narratives illustrated the influence of the assessment culture in shaping the reception and enactment of the renewed assessment plan. Their accounts highlighted how the prevailing cultural milieu across the university influenced the responses of students, faculty and management towards the assessment plan. They described culture in two ways. Firstly, some indicated that to implement the assessment plan, they need a supportive environment where time, resources and manpower are provided. It appeared that these aspects are what constitute a positive learning culture.

I think it takes a lot of time… you also need more people to assist. Not just one person leading the change or the assessment plan, because it’s very different, and because it’s new. And with time, we also need money, you know, with manpower. You need enough team members to facilitate the change, and to put everything into action’ (FGD1: P5).

The idea of a culture that supports the change in assessment was also extended to the need for support in the form of peer feedback and faculty development:

It’s not only students who need feedback. It’s the faculty, for improvement, and for seeing whether you are reaching the learning objectives you want to reach (FGD1: P1).

We need learning support services. So, we need assistance. So I know the faculty is investing a lot in terms of education for assessment and teaching and learning. But I think we need a lot more support in terms of how are rolling out the plan. Centre of Teaching and Learning helped me a lot. If it wasn’t for them, I would still be confused (FGD1: P5).

On the other hand, some mentioned that the new assessment plan has managed to influence the prevailing culture in the institution by changing the way teaching, learning and assessment take place:

And it’s been great listening to colleagues’ minds change, as they are getting exposed to assessment drives learning, we need to choose the correct assessment to drive the learning that we want. I don’t know that we have got it right 100% but they are certainly trying (FGD2: P3).

Ultimately, there was a request for a culture that supports learning and, by implication, supports assessment renewal:

So, there is that whole culture that needs to change, and I think the hidden curriculum and what happens outside of the curriculum also influences it (FGD2:P2).

It was clear that some are prepared to take ownership for changing the culture within the curriculum and across the educational landscape:

So, I think maybe we needed to not only change the perception of students, but also more widely the stakeholders, and even maybe if there is a way that we can speak to the stakeholders where students come from, so that schools also understand that (FGD2: P2).

## Discussion

Developing an appreciation of the experiences of faculty with the new assessment plan is important, as curriculum renewal in higher education is contingent on multiple factors. Harris highlighted some of these factors in the context of medical education, including but not limited to a deficiency in the understanding of the need for change and a lack of commitment from the role-players involved [15].

### The Nature of Change

Notably, the findings from this study revealed a contrast to the potential barriers identified by Harris. For example, faculty expressed an understanding of the nature of change in the way they sought to find meaning in the complexities of this process, and their commitment to working through their challenges in the process of implementing the new assessment plan. Furthermore, there seemed to be a deep commitment to changing the way assessment is done, for example, to critically reflect on current practices and to ensure that these practices are informed by evidence. These findings align with the first two tenets of Fullan’s framework for educational change, wherein he underscores the importance of change leaders acting with a moral purpose and the intention of fostering positive change [16].

### A Commitment to Changing the Purpose of Assessment

A noteworthy finding was that faculty were interested in the ‘assessment for learning’ approach. As mentioned in the introductory section, the shift towards an ‘assessment for learning’ approach represents a significant paradigm shift in medical education, highlighting the use of assessment not just as a measurement of learning, but to actively promote and support it [6]. Harrison et al. suggest that when assessment is integrated with learning, it can, amongst other things, enhance student engagement and promote deeper learning [25].

It was also evident that there is a growing awareness amongst participants about the limitations of traditional assessment methods and an openness to critically examine established practices. This awareness is aligned with current literature, where there is increasing recognition that traditional assessment methods may not adequately capture the complexities of clinical competence [7]. Norcini et al. also emphasise the importance of aligning assessment methods with intended learning outcomes and real-world practice [5]. It is thus critical that faculty in this context are supported to embrace this new way of thinking about assessment. This may be achieved by providing opportunities for ongoing dialogue amongst faculty, students and educational leaders regarding assessment practices and their impact on learning.

### The Need for Collaboration in Taking the Plan Forward

The transition from the previous MBChB assessment plan to the renewed assessment plan presented a significant departure from the traditional approach that faculty were accustomed to. In the past, faculty operated predominantly within subject-specific silos, with each individual responsible for a particular domain. However, the new plan requires cross-disciplinary collaboration, with teams of faculty collectively involved in designing and implementing assessments for specific modules. While this brought opportunities such as collaboration during the implementation process, it also presented various challenges. These included the lack of time, financial resources, additional workload and limited manpower. These experiences are not unique to medical education, where it has been reported that there is a tendency for curriculum developers to commence with overarching goals and principles without considering the resources needed to translate these into teaching and assessment practices [4]. In essence, while the findings highlight the inherent complexities with curriculum renewal in medical education, they simultaneously revealed faculty’s adaptive capacity and collaborative spirit, aligning well with Fullan’s principles of coherence-making, knowledge sharing and relationship building. These are key enablers in navigating the intricacies of assessment renewal. These findings also shed light on the inherent professional learning needs that participants expressed, in terms of curricula renewal, including the incorporation of literature to support practices during assessment renewal, the facilitation of peer feedback and guidance on specific skills related to assessment practices.

### The Need for an Assessment Culture that Supports Change

Faculty in this study argued for the need for a culture where assessment is enhanced. While the organisational structures at the institution provided the support for change and innovation through policy development, it was not clear that this was sufficient, as evidenced by the multiple requests from participants for faculty development. Furthermore, it was difficult to determine whether this support created a culture that drives flexible assessment (as is expected with the renewed assessment plan). In more practical terms, one could view a culture that supports renewal in terms of whether curriculum renewal meets the standards of ‘reality and utility’ [19]. In other words, asking questions like: is the change relevant to dealing with issues that are contextual, authentic and real?

#### Recommendations

This study included a cohort of faculty from multiple disciplines who are involved in implementing the renewed assessment plan. They took it on board, experiencing an onslaught of change. The findings underscore the variety of influences on the implementation of this assessment plan within a resource-constrained setting. It is therefore recommended that institutional leadership critically evaluate the allocation of resources, including financial and human capital, to effectively support and sustain curriculum renewal processes across the institution.

Echoing the recommendations of Maaz et al., robust organisational support in the form of a designated curriculum team is necessary to facilitate and maintain the changes catalysed by the curriculum renewal process [26]. Within resource-constrained environments, where it may not always be possible to have a designated curriculum team, leveraging existing faculty committees or working groups to drive the curriculum renewal process should be considered.

Although participants in the study remained committed, resilient and creative, they emerged as wanting to learn more. Thus, there is a need for faculty development opportunities to enhance assessment literacy and practices. Furthermore, it is recommended that the faculty development team explore all professional learning needs of faculty. By designing and delivering faculty development initiatives that directly address these contextual needs, institutions can optimise the limited resources at their disposal, enhancing the efficacy and impact of capacity-building efforts [27, 28], especially in resource-constrained settings.

Other learning needs, such as faculty’s willingness to engage in peer learning, also emerged prominently amongst the participants. It is therefore recommended that the institution actively cultivates a culture of peer learning as well as mentorship, fostering an environment of shared expertise, mutual support and continuous professional growth.

Echoing the perspectives of Fullan and Land, it is recommended that institutional leadership establish an ongoing culture of change, characterised by a sustained commitment to faculty development [16, 28]. Furthermore, they need to recognise that assessment renewal is an iterative and ongoing process that necessitates continuous capacity building and adaptation. By embedding a culture of change within the institutional fabric, faculty will be empowered to continually refine and enhance their pedagogical practices, ensuring alignment with the evolving curricular landscape and maximising the long-term success of the renewal initiative [29, 30].

## Limitations

There is a possibility that faculty who were more positively predisposed towards the renewed assessment plan were the ones who agreed to participate in the FGDs. This may have resulted in the underrepresentation of dissenting views, creating an incomplete picture of the challenges and barriers. In addition, out of a population of 29, only 10 agreed to participate. While this may be seen as underrepresentation, the diversity of the group in terms of their roles and the rich discussions during the FGD provided valuable insights. To address these limitations, the research team engaged in ongoing discussions and reflexive practices to critically examine how our positions and biases might have influenced the data collection and analysis processes. Future research could also address these limitations by employing mixed methods approaches, including surveys to capture a broader range of faculty perspectives.

## Conclusion

This study aimed to understand the experiences of faculty with the implementation of the renewed assessment plan, with the purpose of informing faculty development initiatives. The findings suggest that while there are various challenges associated with the implementation of the assessment plan, this is a work-in-progress and faculty are invested in this change. Embedded within their experiences is an urgent call for a change in the culture to support assessment renewal, as well as various suggestions for faculty development, adding to the current discourse in this field.

## Data Availability

The data that support the findings of this study are available on request from the corresponding author. The data are not publicly available due to their containing information that could compromise the privacy of the research participants.
